# A standardized decannulation protocol improves outcomes in neurorehabilitation of critically ill patients: a quasi-experimental pre-post design

**DOI:** 10.3389/fneur.2026.1686255

**Published:** 2026-02-04

**Authors:** Liang Zhu, Hong Liu, Na Li, Hongxia Pan, Jianmei Zhang, Hongying Jiang, Chunping Du

**Affiliations:** 1 Rehabilitation Medicine Center, West China Hospital, Sichuan University, Chengdu, Sichuan, China; 2 Key Laboratory of Rehabilitation Medicine in Sichuan Province, Chengdu, Sichuan, China

**Keywords:** ATPPM protocol, decannulation, decannulation rate, neurorehabilitation of critical patients, tracheotomy

## Abstract

**Purpose:**

Clinical practice guidelines recommend tracheotomy decannulation protocols for neurorehabilitation of critically ill patients. However, evidence supporting their effectiveness remains limited. This study aimed to analyze the effect of a standardized decannulation protocol on decannulation rates in tracheotomized patients undergoing neurorehabilitation.

**Methods:**

Participants were recruited from inpatients in the rehabilitation units of a university-affiliated hospital in Sichuan, China. Tracheotomized patients undergoing neurorehabilitation were assigned to the observation and control groups. The observation group received the standardized decannulation protocol (pre-decannulation assessment, training, preparation, decannulation procedure, and post-decannulation monitoring, ATPPM). This protocol is a multidisciplinary rehabilitation bundle based on the principle of optimizing secretion management. The control group received the routine decannulation protocol (pre-protocol) before the implementation of the new protocol. Outcomes included decannulation rates, the interval from capping to decannulation for metallic tracheotomies, tracheotomy duration, length of stay (LOS), and adverse events.

**Results:**

A total of 466 participants were assigned to the control group (*n* = 242) and observation group (*n* = 224). Diagnostic distribution included spinal cord injury (SCI), stroke, and traumatic brain injury (TBI). The age of the control group was 50.59 ± 14.87 years, and that of the observation group was 53.00 ± 15.76 years. The male-to-female ratio of the control group was 2.06:1, and that of the observation group was 3.07:1. The decannulation rate was higher in the ATPPM group than in controls (63/224, 28.12% vs. 49/242, 20.24%, *p* = 0.047). The ATPPM group had a shorter tracheotomy duration compared with controls (73.5 days vs. 89 days). LOS was significantly reduced in the ATPPM group than in controls (27 days vs. 54 days). An adverse event occurred in one patient (1/224, 0.45%) of the ATPPM group: a tracheotomy tube dislodgement requiring prompt reinsertion.

**Conclusion:**

Implementation of the ATPPM decannulation protocol in a specialized, hospital-based neurorehabilitation unit significantly improved decannulation outcomes for inpatients with neurological disorders who were medically stable but tracheotomy-dependent. While these retrospective findings are promising, future prospective studies, randomized controlled trials, are essential to confirm efficacy and control for potential confounding variables.

## Introduction

Tracheotomy is a common surgical operation used in severely ill patients to maintain a long-term airway, typically following prolonged endotracheal intubation. It is commonly used in patients with severe acquired brain injury (e.g., stroke, traumatic brain injury) who require extended mechanical ventilation or have compromised airway protection reflexes due to neurological dysfunction. By bypassing the upper airway, it improves breathing, reduces sedative requirements, and enables more active engagement in rehabilitation (1). Approximately 100,000 tracheotomies are performed annually in the United States (2), and European data indicate tracheotomy rates of 7–16% among neurocritical care patients (3). Tracheotomy rates are higher in specific neurological conditions: 1.3% in severe ischemic stroke (4), 10–43% in severe traumatic brain injury (TBI) (5), and 21–77% in cervical spinal cord injury (SCI) (6). Globally, approximately 250,000 tracheotomies are performed annually in developed countries (7), and in-hospital mortality for this patient population is considerable, ranging from 10 to 60% (8), largely reflecting the severity of their primary illnesses. To improve outcomes for specific high-risk survivors, such as critically ill patients undergoing neurorehabilitation, implementing specialized, structured approaches to decannulation is crucial.

Neurorehabilitation of critically ill patients refers to those who have survived an acute severe neurological insult (e.g., severe stroke, traumatic brain injury, or spinal cord injury) and have been transferred from a neurological or general ICU to rehabilitation wards. These patients are considered stable enough for early rehabilitation but often continue to have significant neurological impairments and require a tracheotomy for prolonged airway protection (9). Rehabilitation of these patients is complicated by the severity and complexity of their underlying disorders, necessitating close medical supervision. Patient outcomes are greatly influenced by the combination of neurological abnormalities, physical limitations, and systemic health issues (10). Thus, decannulation represents a key component of the rehabilitation process.

Tracheotomy is a crucial airway management procedure. Long-term cannulation, however, weakens the respiratory defenses, increasing the risk of recurring respiratory infections, lengthening hospital stays, raising medical costs, and increasing the demand for nurses (11). Patients with neurological conditions present particular challenges since they frequently experience complications such as lung infections (12), have a high tracheotomy rate, and require long cannulation periods. All of these factors combine to make delayed decannulation a significant clinical problem. A crucial rehabilitation milestone is the timing of decannulation; if done too soon, it increases the risk of reintubation, while doing it too late increases the risk of complications and hinders functional recovery. International studies have produced standardized decannulation techniques with partially quantifiable criteria (13, 14). However, China’s expert consensus guidelines lack disease-specific adaptations (15, 16). As a result, the majority of decannulation decisions are still based on empirical evidence. There is currently no integrated protocol that smoothly incorporates pre-decannulation assessment, procedural safety, and post-decannulation monitoring into a single, operationalized care route, and current guidelines are insufficiently precise for this complex population. To address this gap, our institution implemented a standardized decannulation protocol for neurorehabilitation of critically ill patients.

The standardized decannulation protocol that was developed based on the literature (17–19) and clinical practice aimed to standardize the management of tracheotomy patients. The standardized framework (ATPPM protocol) comprised five sequential phases for: (1) pre-decannulation assessment (A); (2) pre-decannulation training (T); (3) pre-decannulation preparation (P); (4) standardized decannulation procedure (P); and (5) post-decannulation monitoring and management (M). The comparative efficacy of the ATPPM protocol is uncertain. So the study was designed to answer the following research questions: (1) What is the level of decannulation rates of tracheotomized patients undergoing neurorehabilitation? (2) Is there a difference in patients’ outcomes based on the ATPPM protocol?

## Methods

### Study design

A quasi-experimental pre- and post-design was implemented. There were two groups of tracheotomized patients undergoing neurorehabilitation. The observation group received the ATPPM decannulation protocol, and the control group received the pre-protocol during admission.

### Study participants

This study enrolled tracheotomized patients undergoing neurorehabilitation admitted to the Rehabilitation Medicine Center of West China Hospital, Sichuan University, between January 2023 and December 2024. Following departmental implementation of the ATPPM decannulation protocol in January 2024, participants were divided into the observation group and control group by admission time.

Patients were eligible as follows: (1) age above18 years; (2) have a tracheotomy; (3) primary neurological diagnosis including stroke, SCI, or TBI; and (4) not ventilator dependent. We excluded patients who were transferred to the ICU and had incomplete clinical documentation.

### Pre-protocol

The control group received the routine decannulation protocol (pre-protocol) based on the doctor’s experience and the standard clinical practice at our institution prior to the implementation of the new protocol. This included (1) a comprehensive evaluation of patients’ respiratory, swallowing, coughing abilities, and stability of the underlying disease; (2) verifying airway safety through the cuff deflation test and gradual blockage test; and (3) using oil gauze and butterfly tape to pull the incision closed after removing the tube.

### ATPPM protocol

The observation group received the ATPPM decannulation protocol. The protocol consisted of five standardized stages (Table 1). From pre-decannulation assessment to post-decannulation monitoring, the entire procedure is covered by this agreement. Briefly, the protocol begins with the “A” phase, in which a multidisciplinary assessment determines whether decannulation is appropriate. The Pre-decannulation training (T) includes the respiratory and swallow function training. Prior to decannulation (P), the team performs final verification and develops emergency plans. Decannulation is then performed at the bedside using standardized procedures (P). Finally, the post-decannulation monitoring phase (M) involves close observation for at least 24 h. The ATPPM protocol is not merely a decannulation checklist but a comprehensive, multidisciplinary rehabilitation bundle. Its foundational principle is that successful decannulation in neurological patients depends on the systematic optimization of secretion management through four targeted pre-decannulation training components.

**Table 1:** ATPPM decannulation protocol of neurorehabilitation critical patient with tracheotomy.

Phase	ATPPM decannulation protocol details
Pre-decannulation assessment (A)	1. Multidisciplinary collaboration (MDT) Establish a specialized team comprising rehabilitation physicians, rehabilitation nurses, rehabilitation therapists, ICU physicians, and otorhinolaryngologists (ENT). Conduct weekly meetings to discuss patient progress toward decannulation and adjust the plan. 2. Standardized decannulation assessment Utilize an MDT-developed tracheotomy decannulation assessment form (Supplementary Table S1). Conduct joint MDT evaluations twice weekly on fixed days (e.g., Monday and Thursday) to formally determine decannulation readiness. 3. Clinical evaluation Assess patient stability: age, stability of primary diagnosis, comorbidities, vital signs, respiratory status, and level of consciousness. Employ individualized consciousness assessment scales based on patient status, including the Glasgow Coma Scale (GCS), Coma Recovery Scale-Revised (CRS-R), and Full Outline of UnResponsiveness (FOUR) score. 4. Decannulation criteria ① Suctioning frequency ≤2 times per 8-h shift for ≥24 consecutive hours. ② Peak Cough Flow >160 L/min. ③ Maximum Expiratory Pressure ≥40 cmH_2_O. ④ The ability to voluntarily and effectively cough secretions out through an uncapped tracheotomy tube. ⑤ Penetration-Aspiration Scale score ≤5 on endoscopic evaluation. ⑥ Bronchoscopic evidence of airway patency (luminal stenosis <50%). These criteria need to be met simultaneously.
Pre-decannulation training (T)	1. Airway management Maintain airway humidification via routine nebulization combined with a heat and moisture exchanger. Perform subglottic suctioning when indicated. Implement a bedside sputum record chart documenting volume, color, and consistency. Monitor cuff pressure dynamically every 4–6 h, maintaining 25–30 cmH_2_O. 2. Swallowing function training Administer transcutaneous electrical stimulation combined with conventional swallowing stimulation exercises twice daily, 30 min per session. 3. Inspiratory muscle training (IMT) Implement threshold IMT combined with transcutaneous phrenic nerve stimulation twice daily, 45 min per session. 4. Position management Maintain head of bed elevation at 30–60°. Utilize the elevate and raise the bed for progressive postural training. Document vital signs during positioning sessions using a dedicated record form.
Pre-decannulation preparation (P)	1. Pre-capping bronchoscopy Perform flexible bronchoscopy 1 week prior to initiating capping trials. 2. Tracheostomy exchange Exchange plastic tracheotomy tube for a metal tracheotomy tube. 3. Intermittent capping trial Initiate a 24-h intermittent capping trial using a standardized tracheotomy tube cap. Continuously monitor respiratory rate (RR) and oxygen saturation (SpO_2_). 4. Continuous capping trial If stable during intermittent capping, proceed to continuous capping on designated days (e.g., Monday, Tuesday, and Friday) with continuous RR and SpO_2_ monitoring. 5. Post-capping bronchoscopy Perform repeat flexible bronchoscopy within 72 h of successful continuous capping. 6. MDT decannulation decision The MDT confirms decannulation readiness based on bronchoscopy findings and the standardized decannulation assessment scale.
Standardized decannulation procedure (P)	1. Performer Decannulation is performed by an ENT physician at the bedside in the rehabilitation ward. 2. Technique Under continuous cardiac monitoring, perform thorough airway suctioning. Follow the standardized sequence: ① Tube removal. ② Stoma disinfection. ③ Application of butterfly traction to the skin edges. ④ Application of sterile gauze pressure dressing. ⑤ Secure fixation with adhesive tape.
Post-decannulation monitoring and management (M)	1. Time-stepped respiratory monitoring Monitor RR and SpO_2_ continuously immediately post-decannulation. Record RR and SpO_2_ every 5 min for the first 30 min, then every 30 min for the subsequent hour, continuing for a total of 24 h. 2. Complication preparedness Establish emergency protocols for potential complications (e.g., laryngospasm, respiratory distress). 3. Stoma care Perform daily stoma site disinfection until complete epithelialization/closure is achieved.

### Outcomes

Researchers retrospectively extracted clinical data from electronic medical records. Outcome measures included: (1) Decannulation rate: defined as maintaining airway patency without reintubation or tracheotomy reinsertion within 72 h. (2) Interval from capping to decannulation (for metallic tracheotomy tubes): the time between capping the tube and its removal. (3) Tracheotomy duration: the time from tracheotomy placement to decannulation (or to rehabilitation discharge for non-decannulation patients). (4) Length of Stay (LOS): total inpatient days in the rehabilitation unit. (5) Adverse Events: including unplanned decannulation, reintubation, post-decannulation respiratory failure, and death during protocol implementation.

### Data analysis

Statistical analysis was performed using SPSS 19.0 software (IBM Corp., Armonk, NY). Continuous variables with normal distribution were expressed as mean ± standard deviation (SD), while non-normally distributed variables were reported as median with interquartile range (IQR). Categorical data were presented as frequencies (percentages). To compare baseline characteristics between groups, we used Fisher’s exact test for categorical variables with expected cell counts <5, Pearson’s chi-squared test for other categorical variables, and independent samples *t*-tests for normally distributed continuous variables. The primary outcome, decannulation rate, was analyzed using chi-squared testing. Secondary continuous outcomes, such as the interval from capping to decannulation for metallic tracheotomy tubes, tracheotomy duration, and LOS, were compared between groups using Mann–Whitney U tests due to non-normal distributions. The incidence of adverse events was analyzed using Fisher’s exact test. A two-sided *p* < 0.05 defined statistical significance for all analyses.

### Ethical considerations

This study was approved by the Ethics Committee of West China Hospital of Sichuan University (No. 2025–1,293) on 1 July 2025. The Clinical trial number was ChiCTR2500105605 registering on 7 July 2025. As the data were sourced from electronic medical records, we have applied for an exemption from informed consent.

## Results

### Characteristics of two groups of patients

**Figure 1:**
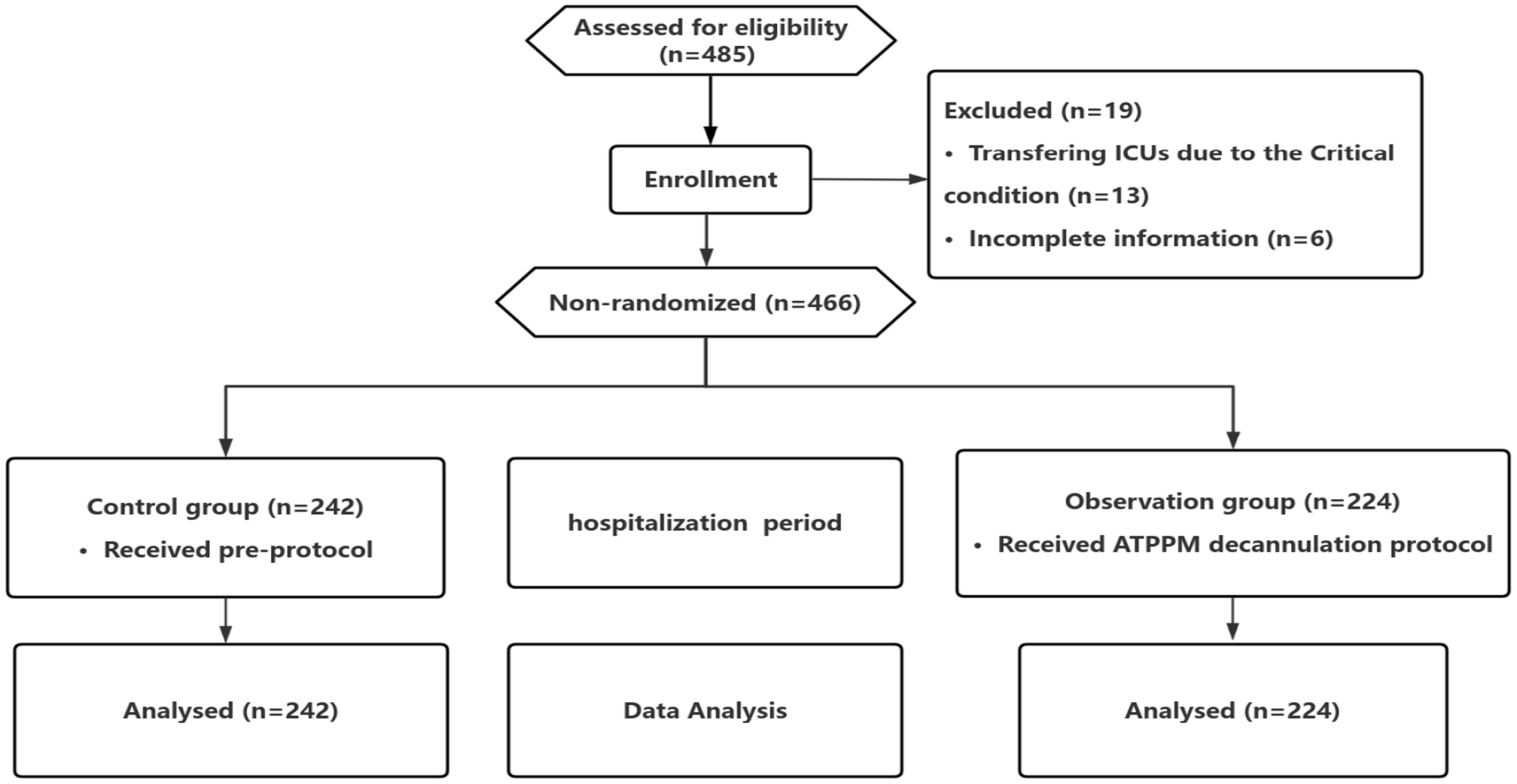
CONSORT flow diagram.

A total of 485 participants were screened before the study, with 466 participants who met the study’s criteria. The study included 242 tracheotomized controls (180 men, 62 women; mean age 50.59 ± 14.87 years) and 224 patients on the ATPPM protocol (169 men, 55 women; mean age 53.00 ± 15.76 years). The control group had 94 cases of SCI, 113 stroke cases, and 35 TBI cases, whereas the observation group had 73 cases of SCI, 98 stroke cases, and 53 TBI cases (Table 2). The reasons for attrition were the critical state and incomplete information (Figure 1).

**Table 2:** Characteristics of the two groups of neurorehabilitation critical patient with tracheotomy.

Variable		Total (*n* = 466)	Control group (*n* = 242)	Observation group (*n* = 224)	*P*
Age (year)—mean (SD)		51.79 ± 15.31	50.59 ± 14.87	53.00 ± 15.76	0.091
Sex	Female	134 (28.76)	79 (32.64)	55 (24.55)	0.054
	Male	332 (71.24)	163 (67.36)	169 (75.45)	
Etiology	Stroke	187 (40.13)	89 (36.78)	98 (43.75)	0.180
	SCI	171 (36.70)	98 (40.49)	73 (32.59)	
	TBI	108 (23.17)	55 (22.73)	53 (23.66)	
Consciousness	Awake	127 (27.25)	65 (26.86)	62 (27.68)	0.394
	Consciousness disorder	85 (18.24)	39 (16.12)	46 (20.53)	
	Unconscious	254 (54.51)	138 (57.02)	116 (51.79)	
Cognitive impairment		322 (69.09)	158 (65.29)	164 (73.21)	0.064
Type of tracheal tube	Metallic	141 (30.26)	69 (28.51)	72 (32.14)	0.394
	Non-metallic	325 (69.74)	173 (71.49)	152 (67.86)	
Electronic laryngoscopy ^a^	Normal	86 (38.91)	41 (40.19)	45 (37.82)	0.389
	Less than 1/4 granulation tissue	87 (39.37)	44 (43.14)	43 (36.13)	
	Less than 1/2 granulation tissue	41 (18.55)	15 (14.71)	26 (21.85)	
	More than half of the granulation tissue	7 (3.17)	2 (1.97)	5 (4.20)	
Payment method	Self-funded	236 (50.64)	128 (52.89)	108 (48.21)	0.313
	Medical insurance	230 (49.36)	114 (47.11)	116 (51.79)	

^a^Not all patients underwent electronic laryngoscopy examination, with 102 in the control group and 119 in the observation group.

### Primary outcome

Successful decannulation was achieved in 49 controls (20.24%) vs. 63 ATPPM-managed patients (28.12%; χ^2^ = 3.953, *p* = 0.047) in Table 3. All decannulated patients maintained airway patency without requiring reintervention.

**Table 3:** Outcomes in two groups of critically ill neurorehabilitation patients with tracheotomy.

Variable	Control group (*n* = 242)	Observation group (*n* = 224)	*P*
Decannulation rate (n/%)	49 (20.24)	63 (28.12)	0.047
Tracheotomy duration (d)	89 (81–98)	73.5 (49–124.75)	0.000
LOS (d)	54 (31–65)	27 (22–35)	0.000
The interval from capping to decannulation for metallic tracheotomy tubes (d)	7 (4–11)	5 (3–8)	0.014

### Secondary outcomes

**Figure 2:**
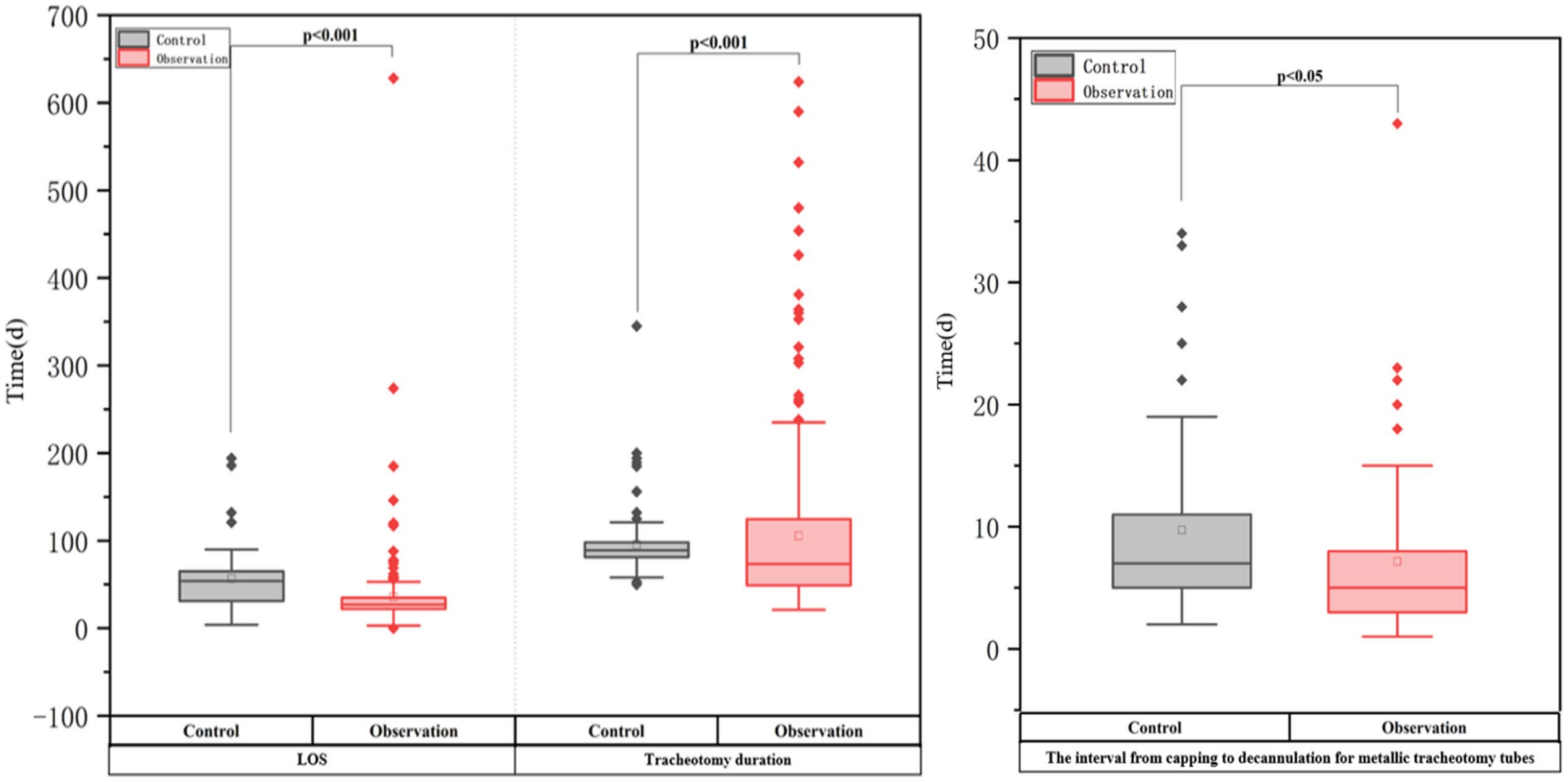
Outcomes in two groups of critically ill neurorehabilitation patients with tracheotomy.

**Figure 3:**
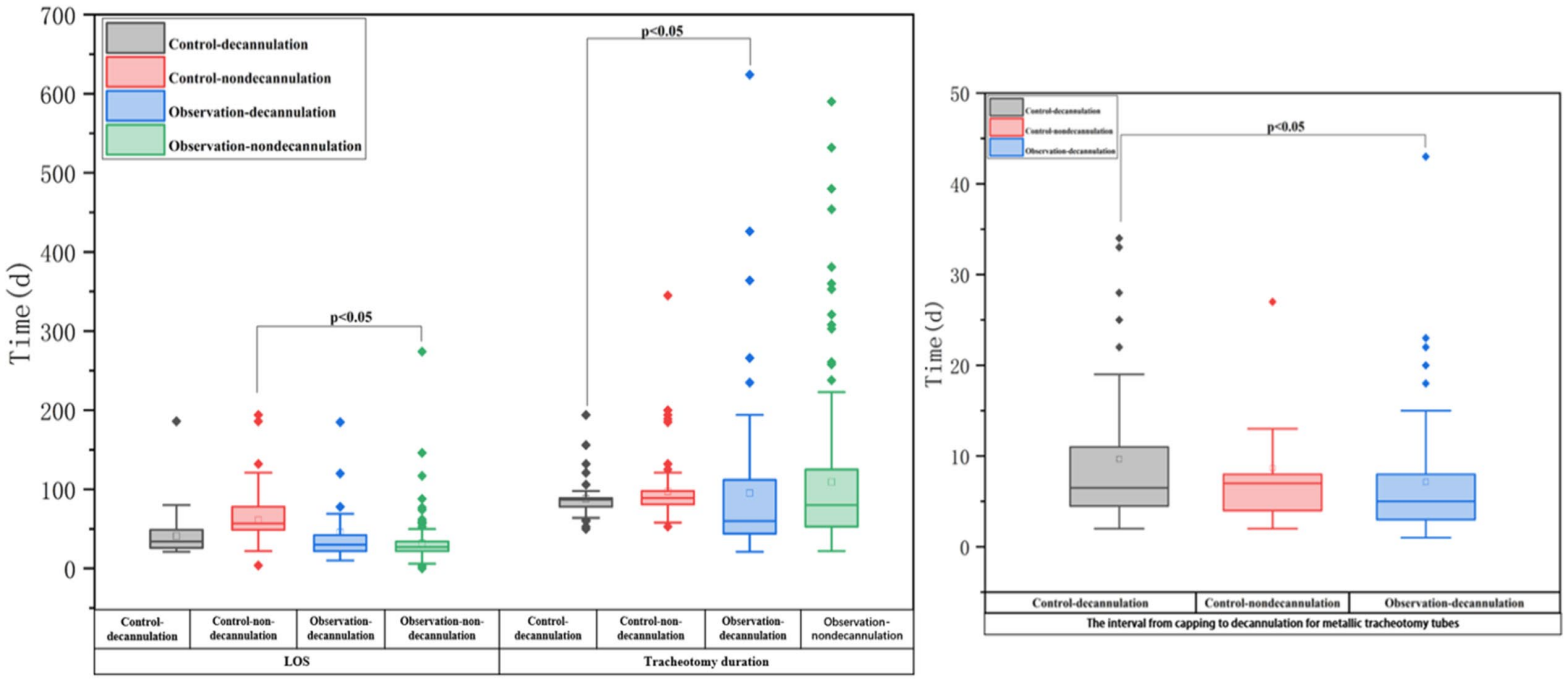
Between-group comparison of secondary outcomes by decannulation vs. non-decannulation.

Table 3 and Figure 2 show that the ATPPM group had significantly shorter tracheotomy duration [median 73.5 days (IQR 49–124.75) vs. 89 days (IQR 81–98); *p* < 0.001] and LOS [median 27 days (IQR 22–35) vs. 54 days (IQR 31–65); *p* < 0.001] than the controls. The interval from capping to decannulation for metallic tracheotomy tubes was shorter in the ATPPM group [median 5 days (IQR 3–8) vs. 7 days (IQR 4–11); *p* = 0.014]. Decannulated patients in the ATPPM group showed reduced intervals compared with controls, as shown in Figure 3 and Supplementary Table S2.

### Adverse events

One patient (1/224, 0.45%) in the ATPPM group experienced a minor adverse event, consisting of an unplanned tracheotomy tube displacement that required prompt reinsertion.

## Discussion

This study found a 28.12% (63/224) tracheotomy decannulation rate in ATPPM-managed neurorehabilitation of critically ill patients, with a median tracheotomy duration of 73.5 days. Decannulation is still contraindicated in some challenging neurological circumstances, as illustrated by the 13 patients who were removed from protocol execution due to clinical instability that required ICU transfer. When compared to the pre-protocol, the ATPPM approach improved decannulation rates. Compared to previous smaller cohort studies, our observed rates are lower, ranging from 35.9 to 52.13% (7, 20, 21). This disparity is because only study focused exclusively on tracheotomized patients undergoing neurorehabilitation who have significant neurological impairments, such as stroke, traumatic brain injury, or spinal cord injury, who also exhibited reduced respiratory function, prolonged comatose states, and impaired airway protective reflexes. Together, these pathologic factors increase the complexity of decannulation and lower the likelihood of success.

Interestingly, our decannulation success rate exceeds the 70–88.79% success rates and 40–60% failure rates reported in the neurosurgical literature (22–25). Compared with controls, the ATPPM procedure significantly shortened the interval from capping to decannulation for metallic tracheotomy tubes. Because neurological patients are particularly vulnerable to aspiration and have impaired secretory clearance, we maintained a uniform 72-h evaluation period, despite the fact that international occlusion trial lengths ranged from 12 to 72 h without consensus (26–29). Patients’ tracheotomy duration and rehabilitative LOS were significantly longer than global norms of 11–20 and 12–46 days, respectively (7). This reflects our cohort’s high acuity, especially among cervical SCI patients (C5 and above) who require ventilator support due to diaphragmatic involvement (30, 31). Our technique complied with early removal guidelines with few lung infections, even though the cannulation times were lengthy (17–19).

One patient (0.45%) in the ATPPM group experienced a minor adverse event, consisting of an unintentional tracheotomy tube dislodgement during routine metallic cannula exchange. This incident was resolved immediately by reinserting the tube without impairing breathing or producing hemodynamic instability. The low incidence of adverse events highlights two important clinical factors and aligns with existing safety standards for decannulation techniques. Because metallic cannulas lack cuff retention devices, extra caution is required during exchanges, representing a device-specific risk. Safety validation of the protocol further supports the ATPPM framework’s suitability for neurorehabilitation settings, demonstrating no serious problems, pneumothorax, tracheovascular fistula, or mortality. Despite the fact that our cohort’s tracheotomy duration was longer than expected by international guidelines, the safety profile remained favorable. These findings support the implementation of structured decannulation protocols in complicated neurological populations, as long as staff obtain competency-based training in device-specific management techniques.

Our findings demonstrate that using the ATPPM protocol, which addresses the multifactorial pathophysiology of decannulation failure in neurological patients through a synergistic, bundled approach, resulted in a significantly higher decannulation success rate in neurorehabilitation of critically ill patients. The protocol initially addresses the primary barriers to safe decannulation: (1) IMT targets cough insufficiency to increase peak cough flow; (2) swallowing therapy reduces aspiration risk; (3) airway clearance techniques (such as nebulization and suctioning) manage viscous secretions; and (4) structured positional management counteracts immobility to facilitate pulmonary drainage. Additionally, the multidisciplinary and systematic nature of the ATPPM protocol makes it more effective than single interventions. Applying multiple evidence-based interventions consistently and synergistically improves outcomes, as seen in critical care for VAP prevention (32–35). The ATPPM facilitates a full and uniform recovery by combining respiratory, swallowing, nursing, and physiotherapy knowledge into a standardized mandatory approach. Its effectiveness is most likely due to its systemic robustness as an imposed interdisciplinary bundle and pathophysiological accuracy in targeting secretion management.

### Strengths and limitations

There are a number of significant limitations to this study.

Despite having stringent enrollment restrictions, its retroactive design increases the risk of selection bias. Additionally, there were consistent differences in how the groups managed decannulation: the ATPPM cohort was given a standardized methodology, while the controls relied on experience-based decision-making rather than formal methods. Due to methodological limitations, it is impossible to draw definite conclusions about the regimen’s effectiveness. Despite these limitations, our study has several strengths. To fill a significant gap in the current recommendations, we developed and implemented an evidence-based decannulation paradigm, which has been validated specifically for tracheotomized patients undergoing neurorehabilitation. Furthermore, the ATPPM protocol improved safety parameters while demonstrating compatibility with real-world rehabilitation settings. Clinical endpoints and process indicators were both included in the thorough outcome assessment.

## Conclusion

This study demonstrates that implementation of the ATPPM decannulation protocol significantly increased decannulation rates to 28.12% among tracheotomized patients undergoing neurorehabilitation, representing a statistically significant improvement over the pre-protocol approach. Furthermore, the ATPPM protocol was associated with better clinical outcomes than typical decannulation practices, including shorter tracheotomy duration, lower rehabilitation LOS, and quicker interval from capping to decannulation for metallic tracheotomy tubes. These findings support the clinical efficacy of the ATPPM framework in optimizing tracheotomy decannulation in patients with complex neurological conditions.

## Data Availability

The raw data supporting the conclusion of this article will be made available by the authors, without undue reservation.
