# Supplementary material for: A standardized decannulation protocol improves outcomes in neurorehabilitation of critically ill patients: a quasi-experimental pre-post design
Source: Front Neurol. 2026 Feb 4;17:1686255. doi: 10.3389/fneur.2026.1686255 (PMC12913058; doi:10.3389/fneur.2026.1686255)
Supplement: Supplementary file 1 [file Table_1.docx]

Supplementary Table S1 MDT-developed tracheotomy decannulation assessment form

| **Performer** | **Items** |
| --- | --- |
| ****Rehabilitation** physicians** | 1.Age  2.Primary disease diagnosis  3.Whether there is any damage to the nervous system  4.Stability of the primary disease  5.Consciousness  6.Tracheotomy time  7.Vital signs  8.Pulmonary CT scan  9.Arterial blood gas (ABG) analysis  10.Comprehensive risk assessment (infections, severe arrhythmia, or hemodynamic instability) |
| **ICU physicians and otorhinolaryngologists** | 1.Oxygen saturation on room air  2.Airway patency  3.Type of tracheostomy tube  4.Capping duration |
| ****R**ehabilitation therapists** | 1.Swallowing function  2.Cough effectiveness and secretion management ability  3.Positioning therapy  4.Respiratory training |
| **Rehabilitation nurses** | 1.Airway humidification  2.Cuff management  3.Secretion management  4.Tracheostomy stoma care  5.Vital signs monitoring post-capping |
| **MDT collective decision-making** | Decannulation or not |

Supplementary Table S2 Detailed comparative data of secondary outcomes by decannulation vs non-decannulationion

| Variable | Control group（n=242） | | Observation group（n=224） | |
| --- | --- | --- | --- | --- |
|  | Decannulation  **(n=49)** | Non-decannulation  **(n=193)** | Decannulation  **(n=63)** | Non-decannulation  **(n=161)** |
| Tracheotomy duration（d） | 88（78-89.5） | 89（81-98） | 60（44-112） | 80（52.5-125.5） |
| LOS（d） | 31（24-47.5） | 57（49-78.5） | 30（22-42） | 27（22-34.5） |
| The interval from capping to decannulation for metallic tracheotomy tubes（d） | 7（4-11） | 7（5.25-18.5） | 5（3-8） | NA |
